# Retrospective analysis of primary extranodal unicentric Castleman disease: a systematic review

**DOI:** 10.3389/fmed.2026.1749931

**Published:** 2026-01-22

**Authors:** Jianing Shen, Yongjun Zeng, Yuan Liu, Nie Xu

**Affiliations:** 1 School of Basic Medical Sciences, Chengdu University of Traditional Chinese Medicine, Chengdu, China; 2 Department of Pathology, Chengdu Integrated TCM and Western Medicine Hospital, Chengdu, China; 3 Department of Oncology, The Research Institute of Integrated TCM & Western Medicine of Chengdu University of Chinese Medicine, Chengdu Integrated TCM and Western Medicine Hospital, Chengdu, China

**Keywords:** diagnosis, extranodal, systematic review, treatment, unicentric Castleman disease

## Abstract

**Background:**

Unicentric Castleman disease (UCD) is a rare lymphoproliferative disorder that typically occurs in lymph node-rich regions. Castleman disease originating in solid organs outside the lymph nodes is extremely rare. Current knowledge is limited to isolated case reports, leaving a lack of systematic evidence to guide diagnosis, management, and prognostic assessment.

**Methods:**

We conducted a systematic review of PubMed-reported cases and series of histologically confirmed of primary extranodal UCD over the past 20 years, summarizing their epidemiological, clinical, pathological, therapeutic features and outcome data.

**Results:**

A total of 99 cases from 83 publications were included. The median age at diagnosis was 44 years, with a male-to-female ratio of 0.77:1. The mean tumor size was 4.9 cm. The most frequent sites were the pancreas (14.1%), adrenal glands (14.1%), skeletal muscle (14.1%), parotid glands (13.1%), and kidneys (9.1%). Hyaline vascular type predominated (80.8%). Surgery was the primary treatment, yielding a 4-year recurrence-free survival rate of 84.6% (95% CI, 0.512–0.959). Lymphoma transformation occurred in 2% of cases. Radiotherapy and glucocorticoids may be effective treatment options.

**Conclusion:**

Primary extranodal UCD displays different anatomic origins but generally carries an excellent prognosis following complete surgical resection. Awareness of its potential for malignant transformation is essential for long-term management.

## Introduction

Castleman disease (CD), also known as angiofollicular lymph node hyperplasia, is a rare and etiologically unclear lymphoproliferative disorder characterized by immune dysregulation and abnormal lymphoid tissue proliferation. The disease was first described by Benjamin Castleman in the 1950s (1) and was later classified into unicentric Castleman disease (UCD) and multicentric Castleman disease (MCD) in the mid-1980s, based on the number and distribution of affected lymph nodes (2).

UCD typically manifests as a localized mass involving a single lymph node or a single lymph node region. It usually progresses slowly and carries a favorable prognosis (3). In contrast, MCD is a systemic lymphoproliferative disorder presenting with generalized lymphadenopathy, significant constitutional symptoms, and laboratory abnormalities, often requiring long-term systemic therapy and carrying a higher recurrence risk and poorer prognosis (4, 5).

Epidemiologically, approximately 6,500–7,700 new CD cases are diagnosed annually in the United States (6), with an estimated incidence of 15.9 per million person-years based on the IMS LifeLink™ database for UCD (7). Despite this, epidemiologic data on UCD remain limited. Its clinical manifestations are often nonspecific and insidious, and many cases are discovered incidentally during imaging studies. Common anatomical sites of UCD correspond to lymph node–rich regions, including the mediastinum (29%), neck (23%), abdomen (21%), and retroperitoneum (17%) (8, 9).

By contrast, primary extranodal UCD, arising from solid organs or soft tissue lacking organized lymph node structures—such as the lungs, liver, pancreas, or kidneys—is exceedingly rare.

Current understanding is largely based on scattered case reports, highlighting a significant gap in systematic evidence regarding its epidemiology, clinical behavior, optimal management, and prognosis. To further elucidate the clinicopathologic spectrum of this entity, we conducted a 20-year retrospective systematic review (2005–2025) of published cases of primary extranodal UCD, aiming to clarify its epidemiological characteristics, clinical presentation, pathological subtypes, treatment strategies, and prognostic outcomes.

## Methods

We performed a systematic search of the PubMed database to identify reports of primary extranodal UCD published between January 2005 and July 2025. Extranodal was defined as arising in solid organs or soft tissues outside organized lymph node structures (examples: lung, liver, kidney, pancreas, skin, central nervous system, ureter). The spleen, Waldeyer’s ring, and thymus were excluded from the extranodal definition.

The PubMed query used was: (“Castleman Disease” [Title] OR “Castleman’s Disease” [Title] OR “Castleman” [Title]) AND (case [Title]), with results limited to the specified date range. Titles and abstracts retrieved by this search were screened in a two-stage process: (1) title/abstract screening to remove clearly irrelevant records; and (2) full-text review to confirm eligibility. Study selection adhered to a standardized screening workflow to ensure reproducibility.

Inclusion criteria were: (1) histopathologically confirmed UCD; (2) lesion strictly confined to an extranodal solid organ or soft tissue site as defined above; and (3) provision of sufficient clinical and diagnostic detail (demographic data, presenting symptoms, lesion location and size, pathological subtype, treatment, and outcome). Exclusion criteria were: (1) MCD; (2) concurrent malignant neoplasm; (3) duplicate reports; or (4) reports lacking essential clinical or pathologic information.

Two investigators independently performed title/abstract screening, full-text review, and data extraction using a predefined data collection form. Extracted items included age, sex, presenting symptoms, lesion site and size, histopathologic subtype, treatment modality, follow-up duration, recurrence, and malignant transformation. Discrepancies at any stage were resolved by discussion between the two reviewers to reach consensus. When classification ambiguity (e.g., early reports with unclear UCD/MCD or extranodal definitions) arose, cases were re-evaluated collaboratively to minimize misclassification bias. Selected articles were compiled for descriptive analysis of epidemiologic, clinical, pathological, therapeutic, and outcome variables.

## Results

**Figure 1:**
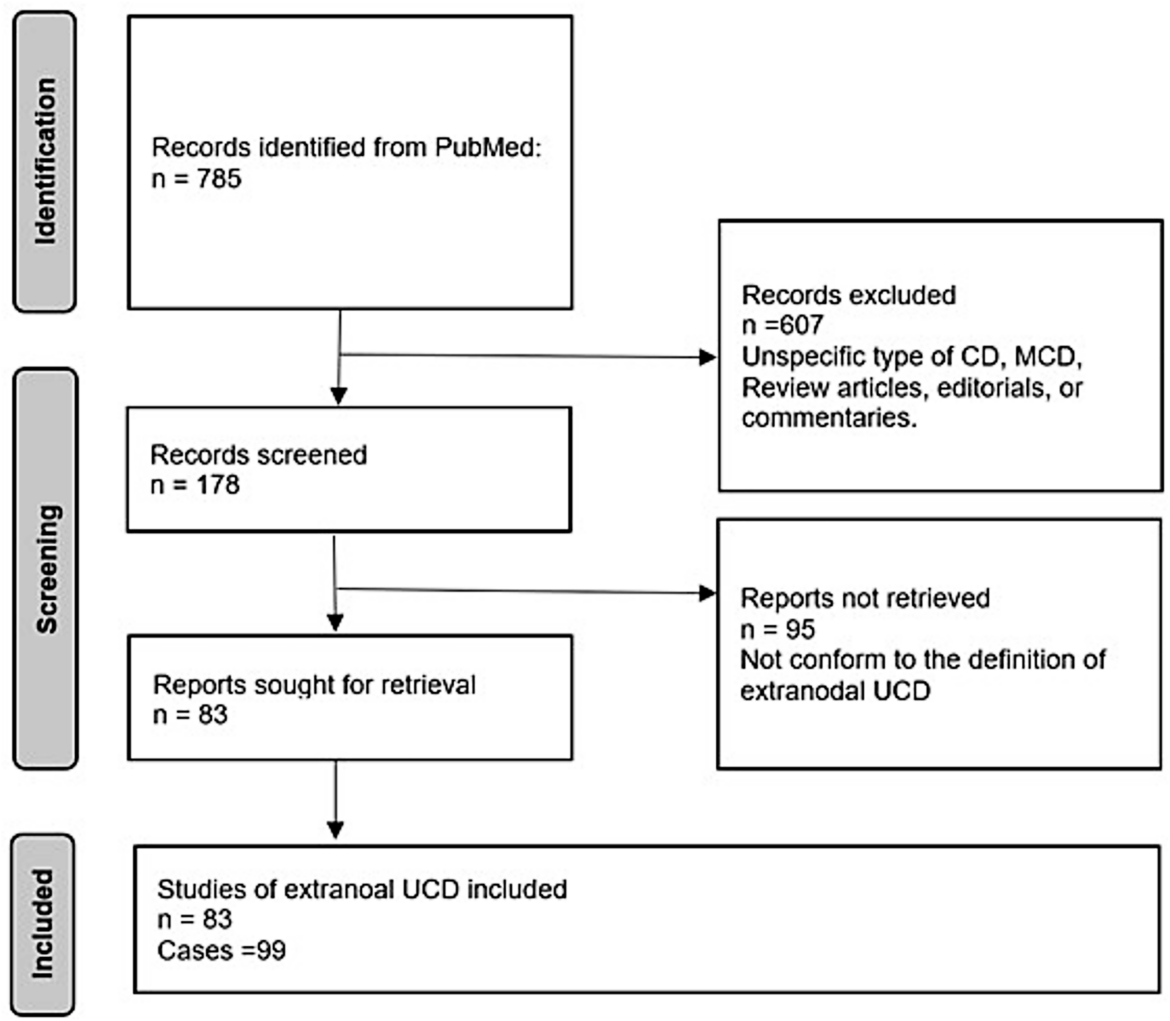
PRISMA diagram investigating resources on extranodal UCD. A total of 785 publications were initially identified through the PubMed search. After title and abstract screening, 178 articles were considered potentially eligible and underwent full-text review. Of these, 95 articles were excluded for not meeting the predefined extranodal criteria. Ultimately, 83 articles were included in the final analysis, encompassing 99 histopathologically confirmed cases of primary extranodal UCD.

A total of 785 publications were initially identified through the PubMed search. After title and abstract screening, 178 articles were considered potentially eligible and underwent full-text review. Of these, 95 articles were excluded for not meeting the predefined extranodal criteria. Ultimately, 83 articles were included in the final analysis, encompassing 99 histopathologically confirmed cases of primary extranodal UCD (9–92). All cases met the completeness criteria for diagnostic and therapeutic data. The literature screening and data extraction were independently performed by two reviewers, with no discrepancies noted. The study selection process adhered to PRISMA guidelines, and the detailed screening flow is illustrated in Figure 1. The clinical and pathological features of the included cases, is detailed in Supplementary material.

### Epidemiological characteristics

As shown in Table 1, the median age at onset of extranodal UCD was 44 years (range 9 to 79 years), with most cases occurring in young and middle-aged adults (20 to 59 years). The number of reported cases has increased over time: 18 cases before 2010 and 81 cases after 2010. The male-to-female ratio was 0.77:1.

**Table 1:** Epidemiological characteristics of extranodal UCD.

Parameters	Subgroups	*N*	%
Total patients		99	
Sex	Male	43	43.4%
	Female	56	56.6%
Age (years)	<20	11	11.1%
	20–39	34	34.3%
	40–59	34	34.3%
	≥60	20	20.2%
Symptoms	None	28	28.3%
	Local compression symptoms	44	44.4%
	General symptoms	6	6.1%
	NA	21	21.2%
Average size (cm)	≤5	59	59.6%
	≤10, >5	26	26.3%
	>10	6	6.1%
	NA	8	8.1%
Histologic variants	HV	80	80.8%
	PC	11	11.1%
	Mixed	5	5.1%
	NA	3	3.0%
Treatment	GCs	2	2.0%
	S + Radiotherapy	3	3.0%
	S + GCs	2	2.0%
	S	91	91.9%
	NA	1	1.0%
Outcome	NED	64	64.7%
	Recurrence and transform into B-cell lymphoma	2	2.0%
	Stable disease	2	2.0%
	NA	31	31.3%

HV, hyaline vascular; PC, plasma cell; NA, not available; GCs, glucocorticoids, S, surgical; SD, stable disease; NED, no evidence of disease; R, recurrence.

### Clinical and pathological features

Of the 99 cases, 44.4% presented with local compression symptoms such as pain or swelling, whereas 28.3% were asymptomatic and discovered incidentally. The average lesion size was 4.9 cm; 59.6% measured ≤5 cm, 26.3% measured 5 to 10 cm, and 6.1% exceeded 10 cm in diameter.

**Figure 2:**
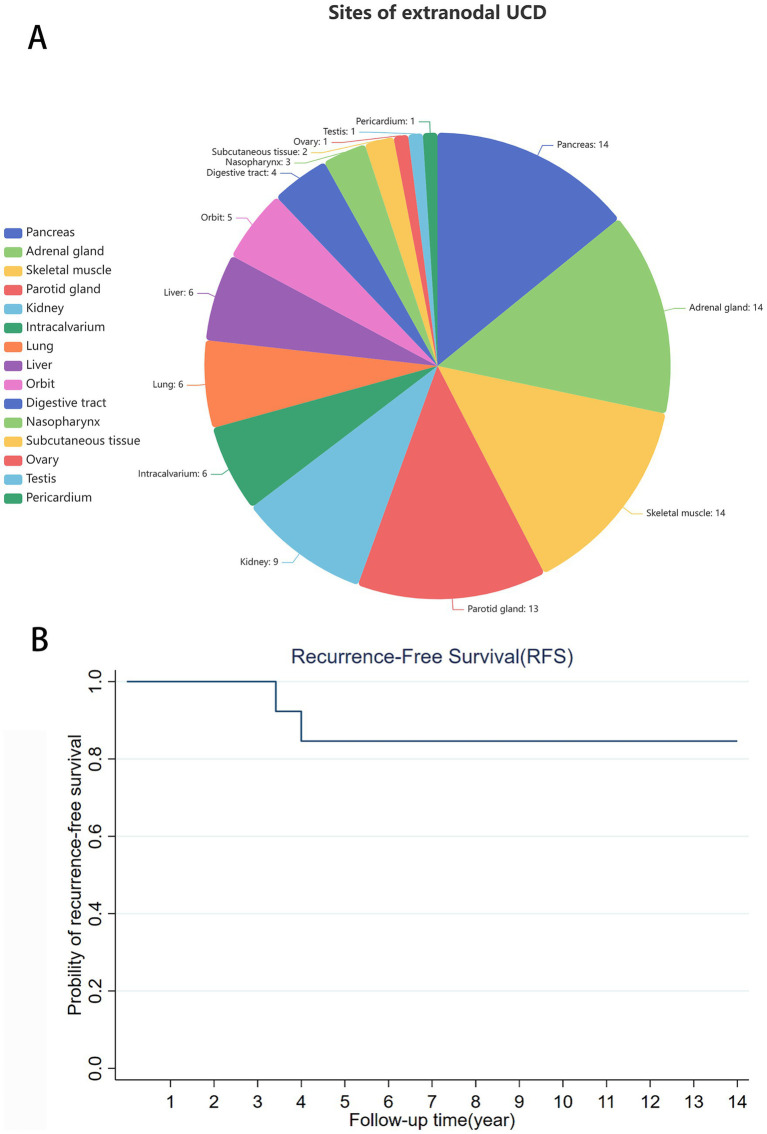
**(A)** The number and proportion of extranodal UCD that occurs in different sites. **(B)** The Kaplan–Meier survival curve of extranodal UCD. The curve started at 100%, with two declines at 3.42 and 4 years, with the recurrence-free survival rate (RFS) of 92.3% at 3.5 years and 84.6% at 4 years. It then plateauing at a high level.

Histologically, 80.8% were classified as HV type, 11.1% as plasma cell (PC) type, and the remainder as mixed or unclassified variants. The most frequently involved extranodal sites were the pancreas (14.1%), adrenal gland (14.1%), and skeletal muscle (14.1%), followed by the parotid gland (13.1%), kidney (9.1%), Intracalvarium (6.1%), lung (6.1%), liver (6.1%), eye (5.1%), digestive tract (4.0%), nasopharynx (3.0%), subcutaneous tissue (2.0%), ovary (1.0%), testis (1.0%), and pericardium (1.0%) (Figure 2A). It’s worth noting that in all the cases included in this study, no cases of TAFRO syndrome were reported. This syndrome is characterized by thrombocytopenia, generalized edema, fever, renal dysfunction and organ enlargement, and is mainly associated with MCD.

### Radiology and laboratory features

In terms of imaging, for superficial lesions, ultrasound is often used as a preliminary examination method. They usually present as clearly demarcated masses with varying internal echoes depending on the composition of the mass, and often show abundant blood flow signals. On CT, the lesions mostly appear as well-defined, uniformly enhanced soft tissue density shadows. Calcification is relatively common and can be present as punctate to large central types, mostly seen in pancreatic, adrenal, and parotid gland lesions. MRI shows different manifestations depending on the location and pathological type, typically showing equal or slightly lower signals on T1-weighted images, equal or slightly higher signals on T2-weighted images, and often showing high signals on DWI, indicating restricted diffusion. A few cases underwent PET/CT examination, which mostly showed increased metabolism. However, for those with obvious calcification, necrosis, or degeneration of tissues, it can lead to uneven FDG uptake or local reduction, affecting the overall metabolic assessment. In summary, the imaging manifestations of UCD lack specificity and are easily confused with malignant tumors. Diagnosis still requires histopathological examination. In terms of laboratory tests, most patients have no significant abnormalities in blood routine and inflammatory indicators, which is consistent with the nature of UCD as a localized disease. Occasionally, there is a slight increase in erythrocyte sedimentation rate, IL-6, and CRP, mostly seen in PC type or mixed type. Patients with systemic symptoms or specific organ involvement may have corresponding abnormal manifestations in their corresponding laboratory tests.

### Treatment and outcomes

Complete surgical resection was the primary treatment modality in 91.9% of cases, while a minority received surgery combined with radiotherapy (3.0%) or surgery combined with glucocorticoid therapy (2.0%). Follow-up data were available for most cases. The median follow-up duration was 14 months, during which 64.7% of patients remained disease-free. The 4-year recurrence-free survival (RFS) rate was 84.6% [95% CI, OR 0.512–0.959], as shown in the Kaplan–Meier survival curve (Figure 2B). Two patients (2.0%) experienced malignant transformation to B-cell lymphoma, with a median transformation time of 45 months (Table 2).

**Table 2:** Different treatment methods and outcomes.

Treatment	*N*	Outcome	*N*
GCs	2	SD	2
S + Radiotherapy	3	NED	3
S + GCs	2	NED	2
S	91	R	2
		NED	59
		NA	30
NA	1	NA	1

S, surgical; NA, not applicable; SD, stable disease; NED, no evidence of disease; R, recurrence.

## Discussion

UCD accounts for approximately 75% of all CD cases, with an estimated annual incidence of 16 to 20 cases per 100,000 population (7). The geographic distribution and histopathological subtype proportions vary considerably across regions. The typical manifestation of UCD is the appearance of a local mass in an area rich in lymph nodes. Talat et al. conducted a large-scale systematic study on 404 published cases of UCD worldwide from 1956 to 2009, finding that 29% occurred in the mediastinum, 23% in the neck, 21% in the abdomen, and 17% in the retroperitoneum (8). Approximately 90% of the UCD cases requiring surgical treatment originated from the lymph node sites. In contrast, primary extranodal UCD is a more rare variant. Our study covered relevant global literature over the past two decades and confirmed only 99 cases confirmed by pathology. This huge difference in the number of cases alone fully demonstrates the rarity of extranodal UCD. Moreover, the anatomical distribution of extranodal UCD is fundamentally different from that within the organ. The cases in this study mainly occurred in solid organs and soft tissues, most of which lacked organized lymph structures. The most common sites were the pancreas, adrenal glands, and skeletal muscles (each accounting for 14.1%), followed by the parotid glands (13.1%) and kidneys (9.1%). This unique distribution leads to the special clinical manifestations of extranodal UCD. The reason for this distribution difference is still unclear and may be related to differences in the pathogenic microenvironment.

The present study systematically reviewed the literature published over the past two decades and identified 99 well-documented cases of extranodal UCD. To our knowledge, this represents the first comprehensive analysis elucidating the epidemiological characteristics, clinicopathological features, and treatment outcomes of UCD arising in extranodal sites.

The pathogenesis of CD remains incompletely understood. For MCD, accumulating evidence implicates viral infection, chronic low-grade inflammation, and cytokine dysregulation (particularly involving IL-6) as major pathogenic mechanisms (93). In contrast, the etiology of UCD is less clear. To date, no specific environmental, racial, or hereditary risk factors have been identified.

Recent molecular pathological studies suggest that UCD, particularly the HV subtype, may represent a monoclonal proliferation derived from lymphoid stromal cells, especially follicular dendritic cells (FDCs) (94, 95). Through whole-exome and targeted deep sequencing, recurrent PDGFRB mutations have been identified as the most common genetic alteration in UCD, indicating that aberrant PDGFRB signaling may contribute to its pathogenesis (96).

UCD can occur across a wide age range, with a median age of approximately 30 years, and demonstrates no significant gender predilection, although some studies have reported a slightly higher prevalence in females (97, 98). UCD may involve lymph nodes in virtually any anatomical region, including the neck, thorax, abdomen, pelvis, axilla, inguinal area, orbit, nasopharynx, and small intestine (3, 99).

In the present analysis of 99 extranodal UCD cases, the median age was 44 years, notably higher than the median age reported in UCD (30 years). Females accounted for 56.6% of the cases, supporting previous observations of a modest female predominance. The most frequently affected extranodal sites included the pancreas, adrenal gland, skeletal muscle, parotid gland, kidney, intracalvarium, lung, and liver, whereas involvement of the eye, digestive tract, nasopharynx, subcutaneous tissue, ovary, testis, and pericardium was relatively rare (Figure 2A). The spectrum of sites underscores the diagnostic challenge, as UCD can mimic primary tumors of these organs.

In this cohort, 44.4% of patients presented solely with localized compression symptoms, such as pain or swelling, while 28.3% were asymptomatic. Systemic manifestations such as fatigue, fever, and weight loss were uncommon, contributing to a high rate of misdiagnosis or delayed diagnosis. In the ureteral case, dull lumbar pain was the initial presentation, and both clinical symptoms and imaging findings were non-specific, making differentiation from urothelial carcinoma, inflammatory granuloma, or metastatic tumors challenging. Therefore, definitive diagnosis relied on histopathological evaluation, with careful differentiation from Hodgkin lymphoma, thymoma, follicular lymphoma, and angioimmunoblastic T-cell lymphoma (1, 3). Furthermore, comprehensive laboratory and imaging assessments are essential to determine the number and distribution of involved lymph nodes and to exclude multicentric disease (100).

Pathologically, Castleman disease can be classified into HV, PC, and mixed subtypes. The HV subtype is the most common, accounting for approximately 65–75% of UCD cases (101). The PC and mixed types exhibit overlapping histological features and are currently grouped as mixed/plasma cell type, comprising 25–35% of UCD cases (102). In this retrospective study, the HV subtype accounted for 80.8% of all extranodal cases, higher than that reported in smaller cohorts, whereas the PC and mixed types represented 11.1 and 5.1%, respectively. Notably, the PC subtype was more frequently associated with systemic manifestations such as fever or fatigue (103).

The hallmark of the HV subtype is abnormal lymphoid follicular hyperplasia accompanied by fibrotic thickening of the capsule and disruption of the lymph node architecture by broad fibrous bands. Within the follicles, atrophic germinal centers composed of depleted B cells are surrounded by concentrically arranged small lymphocytes (“onion-skin” pattern) and hyalinized vessels that traverse the follicles (“lollipop-like” lesion) (6). The interfollicular regions show proliferation of FDCs and plasma cell–like dendritic cells (PDCs), but lack extensive plasma cell infiltration (103, 104). In contrast, the PC subtype is characterized by prominent plasma cell proliferation within the interfollicular zones while largely preserving the overall lymph node architecture. Some PC type cases may exhibit mild vascular proliferation or onion-skin features, but these are less pronounced than in the HV subtype (105).

In this study, 91.9% of patients underwent complete surgical resection, while a minority received surgery combined with postoperative radiotherapy or glucocorticoid therapy. The therapeutic strategy for UCD should be individualized according to the anatomical site, histological subtype, and clinical presentation, as these factors directly influence disease progression, operative risk, and prognosis (3). Surgical excision not only alleviates local compressive symptoms but also facilitates the gradual resolution of associated paraneoplastic syndromes.

Among the reviewed cases, two patients received glucocorticoid-assisted therapy following surgery. Previous reports have suggested that glucocorticoids (GCs) may benefit UCD patients presenting with systemic inflammatory symptoms or severe comorbidities. GCs can alleviate systemic manifestations, reduce lesion volume preoperatively, decrease anesthesia related complications, and suppress postoperative inflammation. Mechanistically, GCs act through glucocorticoid receptor activation, leading to inhibition of lymphoid cell proliferation and induction of apoptosis. However, the precise molecular mechanisms remain incompletely understood, and the therapeutic role of GCs in UCD requires validation in larger cohorts.

Additionally, three patients underwent surgical resection followed by radiotherapy. In two cases, complete excision was infeasible due to tumor adherence to adjacent neural structures; thus, radical postoperative radiotherapy (39.6 Gy in 22 fractions) was administered (17, 57). The third patient received adjuvant radiotherapy (50 Gy) to prevent recurrence (21). The median follow-up duration for these patients was 1.6 years, during which no recurrence was observed, suggesting that surgery combined with localized radiotherapy may improve outcomes in patients with incomplete resection or high recurrence risk.

UCD generally exhibits a favorable prognosis, with a 5-year survival rate exceeding 90% and a low recurrence rate following complete surgical resection (8). In our cohort, only two cases (2.02%) experienced postoperative recurrence during a median follow-up period of 44.5 months, supporting the effectiveness of radical surgical management. The Kaplan–Meier analysis showed that the 3.5-year RFS rate was 92.3% [95% CI, OR 0.566–0.988], the 4-year RFS was 84.6% [95% CI, OR 0.512–0.959], and the 5-year survival rate was slightly lower than previously reported (Figure 2B). Both recurrent cases progressed to malignant B-cell lymphoma. One involved pulmonary HV type UCD, recurring 41 months postoperatively, and the other was parotid HV type UCD, recurring 48 months after surgery. Although rare, such malignant transformation highlights the importance of long-term follow-up (at least 5 years) with periodic monitoring of LDH, *β*₂-microglobulin, and imaging findings to enable early detection of malignant evolution.

Overall, extranodal UCD demonstrates a good prognosis, but the risk factors for malignant transformation remain unclear. They may involve anatomical location, age at onset, or extent of surgical resection, yet further validation is required in large, multicenter studies.

## Limitations

This study has the following limitations. Firstly, the conclusions of this study are mainly based on retrospective case reports, which may be subject to publication bias. That is, cases with good outcomes or special manifestations are more likely to be reported, which may affect the accurate assessment of the surgical cure rate and long-term risks. Secondly, due to the rarity of the disease, the included cases have a lower level of quality, and there is a lack of prospective or controlled study data. The treatment options are mostly based on clinical experience, so the comparison of different intervention measures needs to be interpreted with caution. Thirdly, the follow-up time of most cases is limited, and it cannot rule out the possibility of late recurrence and malignancy of UCD. The long-term risk assessment is still insufficient, highlighting the necessity of extending the follow-up period. Despite these limitations, this study integrates the most extensive case data in this field to provide important structured knowledge for the clinical management of this rare entity. In the future, international multi-center cooperation is needed to establish a prospective patient registration system to provide higher-quality evidence.

## Conclusion

This systematic review provides the first comprehensive summary of the epidemiology, clinicopathological characteristics, and treatment outcomes of primary extranodal UCD. Our findings confirm that complete surgical resection remains the treatment of choice, yielding an excellent prognosis. However, the potential for malignant transformation, although rare, should not be overlooked. Therefore, establishing a standardized, long-term follow-up protocol is essential to ensure early identification and management of recurrence or malignant progression.

## Data Availability

The original contributions presented in the study are included in the article/Supplementary material, further inquiries can be directed to the corresponding author.
