# Supplementary material for: Retrospective analysis of primary extranodal unicentric Castleman disease: a systematic review
Source: Front Med (Lausanne). 2026 Jan 22;13:1749931. doi: 10.3389/fmed.2026.1749931 (PMC12872552; doi:10.3389/fmed.2026.1749931)
Supplement: Supplementary file 1 [file Table_2.DOCX]

**Supplementary table :Data sets of extranodal UCD**

|  | Author | Year | Sex | Age | Site | Symptoms | Size(cm) | Histology | Treatment | Outcome（m） |
| --- | --- | --- | --- | --- | --- | --- | --- | --- | --- | --- |
| 1 | Goetze, O. et al | 2005 | M | 53 | Pancreas | Renal colic | 5.5×4.5 | HV | S | NED(24) |
| 2 | Matsumura, K. et al | 2005 | F | 68 | Intracalvarium | Initiative loss, incoherent speech,  headache, and left hemiparesis | 0.7 | HV | S | NED(36) |
| 3 | Pan, D. L. et al | 2005 | M | 30 | Adrenal gland | Dental ulcer and rash | 7×6×3 | HV | S+GCs | NED(6) |
| 4 | Shiozaki, A. et al | 2005 | F | 29 | Stomach | Upper abdominal pain | 7.7×5.1×4.3 | HV | S | NED(NA) |
| 5 | Akdogan, O. et al | 2006 | F | 52 | Parotid gland | Swelling | R1.1×1.1,L1.5×1.5 | HV | S | NED(6) |
| 6 | Kim, K. N. et al | 2006 | F | 62 | Esophagus | None | 3×3×7 | HV | S | NA |
| 7 | Chang, Z. P. et al | 2007 | M | 64 | Chest wall | Subcutaneous mass | 16×13×10 | HV | S | NED(15) |
| 8 | Finn, M. A. et al | 2007 | F | 19 | Paraspinal region | Cough and nausea and vomiting | 6.2×3×6.8×3×6.1 | HV | S+R | NED(12) |
| 9 | Hata, T. et al | 2007 | M | 52 | Rectum | Epigastric discomfort and tarry stool | 4.0 | PC | S | NED(22) |
| 10 | Hatano, K. et al | 2007 | M | 70 | Kidney | None | 2.0 | HV | S | NED(8) |
| 11 | Krawczun, G. A. et al | 2007 | F | 40 | Lung | Chest pain | 3 | HV | S | NA |
| 12 | Mallik, A. A. et al | 2007 | F | 53 | Intracalvarium | Headache | NA | PC | S+R | NED(24) |
| 13 | Tunru-Dinh, V. W. et al | 2007 | F | 23 | Pancreas | Abdominal pain | 8.0 | HV | S | NA |
| 14 | Caselli, E. et al | 2008 | M | 16 | Parotid gland | Painless swelling | 4.3×3.8×1.9 | HV | S+GCs | NED(24) |
| 15 | Coca, S. et al | 2008 | M | 42 | Intracalvarium | Slight left hemiparesis | NA | Mixed | S | NED(60) |
| 16 | Just, P. A. et al | 2009 | M | 59 | Pericardium | None | 4.5×3.5×3.5 | Mixed | S | NED(6) |
| 17 | Korukluoglu, B. et al | 2009 | F | 49 | Duodenum | Abdominal discomfort and nausea | 4.2×4×4.5 | HV | S | NA |
| 18 | Zhu, Y. C. et al | 2009 | F | 76 | Kidney | None | 5.5 | PC | S | NED(24) |
| 19 | Hakozaki, M. et al | 2010 | F | 28 | Deltoid | Subcutaneous mass | 5.8×4.7×3.7 | HV | S | NED(36) |
| 20 | Jáñez, L. et al | 2010 | M | 69 | Orbit | None | 1.5×3×1.4 | HV | S | NED(NA) |
| 21 | Mahmood, N. et al | 2010 | M | 19 | Parotid gland | None | 3×4.5 | HV | S | NA |
| 22 | Schaefer, I. M. et al | 2011 | F | 37 | Lower limb | Subcutaneous mass | 8.5 | Mixed | S | NED(3) |
| 23 | Schulte, K. M. et al | 2011 | F | 38 | Adrenal gland | Fatigue and joint aches | 4.5×3.5×2.5 | HV | S | NED(36) |
| 24 | Brubaker, J. W. et al | 2011 | M | 53 | Orbit | Subcutaneous mass | 1.5 | HV | S | NA |
| 25 | Reece, B. et al | 2012 | F | 34 | Parotid gland | Left ear tinnitus | 15×15×10 | HV | S | NED(8) |
| 26 | Xu, X. et al | 2012 | F | 38 | Pancreas | Mild chest pain | 3×3 | PC | S | NED(6) |
| 27 | Jang, S. M. et al | 2012 | M | 64 | Kidney | Microscopic hematuria | 4×2.5 | PC | S | NED(3) |
| 28 | Saghafi, H. et al | 2013 | M | 27 | Parapharyngeal space | Pharyngeal mass and deterioration of snoring | NA | HV | GCs | SD(60) |
| 29 | Clain, J. B. et al | 2013 | F | 40 | Parapharyngeal space | Mild symptoms while swallowing | 2.0 | HV | S | NED(1) |
| 30 | Miyoshi, H. et al | 2013 | F | 70 | Liver | None | 2.0 | HV | S | NED(NA) |
| 31 | Turek, G. et al | 2013 | M | 29 | Intracalvarium | Headache | 6×3 | PC | S | NED(120) |
| 32 | Zhao, H. N. et al | 2013 | M | 34 | Nasopharynx | Nasal congestion | 4.1×3.1×2.8 | HV | S | NED(24) |
| 33 | Eszes,N et al | 2014 | F | 51 | Lung | Cough and hemoptysis | NA | PC | S | NED(36) |
| 34 | Liu, Y. et al | 2014 | M | 33 | Lung | None | 3.1×2.9×3.0 | HV | S | NED(24) |
| 35 | Rasheed, A. et al | 2014 | F | 30 | Scapular region | Subcutaneous mass | 3.5 | HV | S | NED(12) |
| 36 | Bollig, C. et al | 2014 | M | 14 | Parotid gland | Subcutaneous mass | 1.9×2.1×3.3 | HV | S | NA |
| 37 | Cui, J. et al | 2014 | M | 33 | Lung | None | 5.5×4.5×3.0 | HV | S | Recurrence after 41months,  then transform into B-cell lymphoma |
| 38 | Hatanaka, K. et al | 2014 | F | 75 | Ovary | None | 2.5 | HV | S | NA |
| 39 | Iaconetta, G. et al | 2014 | F | 35 | Parotid gland | Painless swelling | 4.0×3.0 | HV | S | NED(84) |
| 40 | Jones, N. W. et al | 2014 | F | 17 | Orbit | Painless swelling | 2.5×2.1×1.2 | HV | S | NED(10) |
| 41 | Koh, Y. et al | 2014 | M | 61 | Kidney | None | 1.8×2.0×1.4 | HV | S | NED(6) |
| 42 | Kumar, S. et al | 2014 | M | 15 | Parotid gland | Subcutaneous mass | 3×4 | HV | S | NED(48) |
| 43 | Mukherjee, B. et al | 2014 | M | 48 | Orbit | Subcutaneous mass | NA | HV | NA | NA |
| 44 | Kang, D. et al | 2015 | M | 53 | Lid | None | 3.2×2.6×1.3 | HV | S | NED(3) |
| 45 | Rawashdeh, B. et al | 2015 | F | 16 | Lung | Non-productive cough and chest pain | 4.8 | NA | S | NA |
| 46 | Abo-Alhassan, F. et al | 2015 | F | 29 | Parotid gland | Painless swelling | 4.9×2.8×3.4 | HV | S | NED(NA) |
| 47 | Wang, S. et al | 2016 | F | 39 | Pancreas | NA | 6 | HV | S | NED(80) |
|  |  | 2016 | M | 30 | Liver | NA | 6 | HV | S | NED(61) |
|  |  | 2016 | F | 31 | Adrenal gland | NA | 6 | HV | S | NED(59) |
|  |  | 2016 | F | 24 | Pancreas | NA | 7.5 | HV | S | NED(22) |
|  |  | 2016 | M | 52 | Adrenal gland | NA | 7.5 | HV | S | NED(20) |
|  |  | 2016 | F | 58 | Pancreas | NA | 4.2 | HV | S | NED(40) |
| 48 | Carolus, A. et al | 2018 | M | 51 | Arm | Right upper arm swollen | 11×4 | HV | S+R | NED(18) |
| 49 | Chen,J. et al | 2018 | F | 26 | Adrenal gland | Left flank pain | 4×3 | HV | S | NED(12) |
| 50 | Foster, C. H. et al | 2018 | M | 25 | Paraspinal region | Left flank pain | 5.9×4.1×6.0 | HV | S | NED(7) |
| 51 | Hanamura, T. et al | 2019 | F | 9 | Back | Subcutaneous mass | 3.9×2 | HV | S | NED(7) |
| 52 | Markovic, V. et al | 2019 | M | 44 | Pancreas | Upper abdominal pain | 4.3×3.8×3.4 | HV | S | NED(NA) |
| 53 | Escribano Paredes, J. B. et al | 2019 | M | 30 | Intracalvarium | Generalized tonic-clonic seizure | 2.0 | HV | S | NED(12) |
| 54 | Ferreira Junior, E. G. et al | 2019 | F | 34 | Pancreas | Abdominal pain | 4.0 | HV | S | NED(1) |
| 55 | Gülmez, S. et al | 2019 | F | 38 | Pancreas | Abdominal pain | 4.0×2.0 | HV | S | NA |
| 56 | Chen,B et al | 2020 | M | 25 | Temporalis muscle | Subcutaneous mass | NA | NA | S | NA |
| 57 | Fuse, M.et al | 2020 | F | 46 | Kidney | Left low back pain and discomfort | 3.7×3.5×3.5 | HV | S | NED(12) |
| 58 | Lv, K. et al | 2020 | F | 68 | Liver | None | 1.5 | HV | S | NA |
|  |  | 2020 | F | 56 | Liver | Abdominal pain | 3.5 | HV | S | NA |
|  |  | 2020 | F | 64 | Liver | None | 1.6 | HV | S | NA |
|  |  | 2020 | F | 68 | Liver | None | 2.3 | HV | S | NA |
| 59 | Huang, Z. et al | 2020 | M | 44 | Pancreas | None | 4.0×2.9 | HV | S | NED(36) |
| 60 | Wang, P. et al | 2021 | M | 62 | Kidney | None | 8.2×7.0×10.5 | HV | S | NA |
| 61 | Nishimura, M. F. et al | 2021 | F | 12 | Gluteal region | Local pain | 5.0 | HV | S | NA |
|  |  | 2021 | F | 69 | Kidney | None | 2.0 | HV | S | NA |
|  |  | 2021 | M | 46 | Back | Subcutaneous mass | 4.0 | HV | S | NA |
|  |  | 2021 | M | 14 | Arm | Subcutaneous mass | 3.0 | HV | S | NA |
|  |  | 2021 | F | 52 | Arm | Subcutaneous mass | 5.0 | HV | S | NA |
| 62 | Guo, Z. et al | 2021 | F | 20 | Parotid gland | Subcutaneous mass | 2.5×3.5 | HV | S | NED(12) |
| 63 | Mindiola-Romero, A.E. et al | 2021 | F | 70 | Adrenal gland | abdominal fullness | 7.2×4.0×3.8 | PC | S | NED(6) |
| 64 | Chen,C. H.et al | 2022 | F | 56 | Kidney | Limb edema,shortness of breath,dizziness | 1.3×1.3×1.1 | HV | S | NED(17) |
| 65 | Liu, S. L. et al | 2022 | F | 28 | Pancreas | None | 3.5×3.0 | HV | S | NED(6) |
| 66 | Maghsoudi, R. et al | 2022 | F | 29 | Adrenal gland | Abdominal pain | 5.0×4.5 | HV | S | NA |
| 67 | Song, Z. et al | 2022 | M | 79 | Scrotum | Subcutaneous mass | 7×6×5 | PC | S | NA |
| 68 | Wang, K. et al | 2022 | M | 60 | Kidney | abdominal discomfort | 1.8 | HV | S | NED(NA) |
| 69 | Dickman,J.et al | 2023 | F | 11 | Temporal region | Subcutaneous mass | 3.4×1.4×3 | HV | S | NA |
| 70 | Yu, H. et al | 2023 | M | 58 | Adrenal gland | None | 10×8×5 | PC | S | NED(12) |
|  |  | 2023 | M | 55 | Adrenal gland | None | 6×5×3 | HV | S | NED(48) |
|  |  | 2023 | F | 45 | Adrenal gland | None | 6×4.5×4 | HV | S | NED(144) |
|  |  | 2023 | F | 26 | Adrenal gland | None | 7.5×6×5 | HV | S | NED(196) |
| 71 | Aldosari, S. et al | 2023 | F | 64 | Adrenal gland | None | 11×7×3.5 | Mixed | S | NA |
| 72 | Dev, S. et al | 2023 | F | 46 | Pancreas | None | 3.0×4.1×3.9 | HV | S | NA |
| 73 | Gou, H. et al | 2023 | M | 46 | Pancreas | Abdominal pain | 6.1×4.3 | HV | S | NED(13) |
| 74 | Ahuja,S et al | 2024 | F | 27 | Gluteal region | Subcutaneous mass | 5×4 | HV | S | NA |
| 75 | Rizzo, S. et al | 2024 | F | 60 | Gluteal region | None | 6.5×3.3×2.1 | HV | S | NED(2) |
| 76 | Zhang, Y. et al | 2024 | M | 54 | Parotid gland | Painless swelling | 2×3 | HV | S | NED(8) |
| 77 | Deng, Q. et al | 2024 | F | 44 | Adrenal gland | None | 2.4×3.5 | HV | S | NED(6) |
| 78 | Moguel, A. E. R. et al | 2024 | F | 52 | Intracalvarium | Headaches and language disturbances | NA | PC | S | NA |
| 79 | Pedicelli, A. et al | 2024 | F | 27 | Parotid gland | Painless swelling | 5.0 | HV | S | NA |
| 80 | Ran, D. et al | 2024 | M | 44 | Adrenal gland | None | 6.5×4.5×5.0 | HV | S | NA |
| 81 | Konlack Mekontso, J. G. et al | 2025 | M | 46 | Pancreas | Epigastric pain, weight loss and  obstructive jaundice | 3.2×3 | Mixed | GCs | SD with complete resolution  of symptoms (8) |
| 82 | Pannu, M. K. et al | 2025 | F | 42 | Lung | Persistent cough and fever | 11×8×5 | HV | S | NED(12) |
| 83 | Vinjamur Rajagopal, S. et al | 2025 | M | 30 | Parotid gland | Painless swelling | 3.5×3.7×4 | HV | S | NED(12) |
| 84 | Yuen, K. F. et al | 2025 | M | 21 | Parotid gland | Subcutaneous mass | NA | NA | S | Recurrence after 48months,  then transform into B-cell lymphoma |

Abbreviation: NA: Not applicable; NED: No Evidence of Disease; HV: Hyaline vascular; PC: Plasma cell; S:Surgical; GCs: Glucocorticoids; SD: Stable disease; R:Radiotherapy; m: month.
